# Shorter GT repeat polymorphism in the heme oxygenase-1 gene promoter has protective effect on ischemic stroke in dyslipidemia patients

**DOI:** 10.1186/1423-0127-17-12

**Published:** 2010-02-23

**Authors:** Chyi-Huey Bai, Jiunn-Rong Chen, Hou-Chang Chiu, Chia-Chi Chou, Lee-Young Chau, Wen-Harn Pan

**Affiliations:** 1 Central Laboratory, Shin Kong WHS Memorial Hospital, Taipei, Taiwan; 2 School of Public Health, Taipei Medical University, Taipei, Taiwan; 3 Changhua Christian Hospital Yunlin Branch, Yun-Lin County, Taiwan; 4 Department of Neurology, Shin Kong WHS Memorial Hospital, Taipei, Taiwan; 5 Department of Internal Medicine, Chang Gung Memorial Hospital, Keelung, Taiwan; 6 Institutes of Biomedical Sciences, Academia Sinica, Taipei, Taiwan

## Abstract

**Background:**

The microsatellite polymorphism of heme oxygenase (HO)-1 gene promoter has been shown to be associated with the susceptibility to ischemic event, including coronary artery disease (CAD), myocardial infarction, and peripheral vascular disease. We aimed to examine whether the length of (GT)_n _repeats in HO-1 gene promoter is associated with ischemic stroke in people with CAD risk factors, especially low level of HDL.

**Methods:**

A total of 183 consecutive firstever ischemic stroke inpatients and 164 non-stroke patients were screened for the length of (GT)_n _repeats in HO-1 promoter. The long (L) and short (S) genotype are defined as the averaged repeat number >26 and ≦26, respectively.

**Results:**

Stroke patients tended to have more proportions of hypertension, diabetics and genotype L, than those of genotype S. Patients with genotype L of HO-1 gene promoter have higher stroke risk in comparison with genotype S especially in dyslipidemia individuals. The significant differences on stroke risk in multivariate odds ratios were found especially in people with low HDL-C levels.

**Conclusions:**

Subjects carrying longer (GT)_n _repeats in HO-1 gene promoter may have greater susceptibility to develop cerebral ischemic only in the presence of low HDL-C, suggesting the protective effects in HO-1 genotype S in the process of ischemic stroke, particularly in subjects with poor HDL-C status.

## Background

Heme oxygenese (HO) is a rate-limiting enzyme in heme degradation, leading to the liberation of free iron, carbon monoxide (CO) and biliverdin[1]. HO-1, one of HO isoforms. is a stress-responsive protein induced by various oxidative agents[1,2]. Over past few years, numerous studies have revealed the important function of HO-1 in cardiology by aspects such as inflammation, antioxidant function, apoptosis, hypoxia and ischemia/reperfusion injury, and angiogenesis [3].

HO-1 as a cytoprotective defense mechanism against oxidative insults is through the antioxidant activities of biliverdin and its metabolite, bilirubin [4], as well as the anti-imflammatory, antifibrinolytic and vasodilative actions of CO [2,5,6]. HO-1 also is up-regulated during cerebral ischemia [7-10], in relation to the severity of brain injury [11] or aneurysms [7], and also exert a protective effect on neuronal cell against oxidative stress [12,13]. The first case of HO-1 deficiency in human was identified in 1999 [14], the patient suffered persistent hemolytic anemia and abnormal coagulation/fibrinolysis system associated with elevated thrombomodulin and von Willebrand factor, indicating persistent vascular injury. Two studies focused HO-1 mocroglia/macrophage and cerebrovascular disease speculated the prolong expression of HO-1 in traumatic brain injury, cerebral infarction and aneurysms [7,10]. HO-1 is also induced in atherosclerotic lesions of human and experimental animals, and has a protective role in the blood vessel wall during atherogenesis [15,16]. Overexpression of HO-1 in arterial walls reduces lesion formation as well as intimal hyperplasia subsequent to vascular injury, supporting its vasoprotective function [17-19]. Several positive physiological effects exerted by HO-1 as anti-inflammatory and cytoproective functions in cardiovascular and peripheral vascular disease [20].

The human HO-1 gene is mapped on chromosome 22q12 with a (GT)_n _dinucleotide repeat polymorphism in the proximal promoter region [21,22]. It has been shown that the (GT)_n _repeat is highly polymorphic and modulates the transcriptional activity of HO-1 gene [23,24]. Promoter containing longer (GT)_n _repeats has lower transcriptional activity in vascular cells [24]. We and others have reported that human subjects carrying longer (GT)_n _repeats have increased susceptibility to the development of coronary artery disease [4,24,25], post-angioplasty restenosis [26-28] and advanced peripheral artery disease [29], indicating that HO-1 promoter polymorphism is likely to act as an candidate in the genetic determinant involved in vascular disease.

Ischemic stroke is a common disease with high mortality rate in populations [30], earlier studies have revealed the family history as an independent risk factor, suggesting the involvement of genetic components in the pathogenesis of ischemic stroke [31]. Ischemic stroke shares many common risk factors with other vascular disease, such as hypertension, diabetes, hyperlipidemia and smoking. Although the neuroprotective effect and the ability of reduced infarct size of HO-1 have been shown [32], the only study focused on recurrent and first ischemic cerebrovascular events still not reported a significant association between HO-1 promoter polymorphism and stroke [3]. In view of the vital role of HO-1 in vascular protection, here we aimed, especially in those stroke patients with no history of cerebro- or cardio-vascular events, to examine the association between the risk of ischemic stroke and the length of the (GT)_n _repeats of the HO-1 gene promoter under several vascular conditions: hypertension, diabetes, lipids abnormality and smoking. We also aimed to explore the interaction of the HO-1 genotype and above risk factors on ischemic stroke.

## Methods

### Participants

A total of 183 consecutively hospitalized first-ever ischemic stroke (IS) inpatients and 164 non-stroke (NS) outpatients were recruited from neurological ward and clinics of Shin Kong WHS Memorial Hospital in Taipei city from mar 1996 to Dec 1999. These first-ever inpatients were recruited within the first 48 h (20.3 ± 14.9 h) of the stroke onset. Inclusion criteria for IS patients were: (a) IS patients admitted within 48 hours of onset; (b) age greater than 40 years; (c) no prior history of stroke and myocardial infarction. NS outpatients are those with complaints of nonspecific symptoms such as peripheral vestibulopathy, radiculopathy, low back pain, insomnia, Parkinson's disease, myalgia, arthralgia, muscle pain, muscle stiffness, or headache. Neurologist had confirmed that these NS patients had no evidence of stroke and myocardial infarction. The controls were enrolled during the same recruitment periods with cases, and the recruitment was performed blindly with respect to patient's clinical data and HO-1 genotypes. The study was approved by Ethics Committee/Institutional Review Board (EC/IRB) of the hospital, and informed consent was obtained from every subject.

### Data Collection and measurement

Information on age, sex, residential area, and risk factors of stroke was obtained via interview within 3 to 7 days of admission. Diagnosis of stroke and stroke subtype of each subject was confirmed by a single neurologist (the second author) based on data from clinical assessment and neurological images such as computerized tomography (CT), and other studied tests. Cerebral infarction was defined as a focal neurological deficit of sudden onset that persisted beyond 24 hours in surviving patients with indication of the presence of infarction and the absence of hemorrhage, which was documented by brain CT or by MRI. Information on medication and on chronic diseases such as hypertension, diabetes mellitus, coronary heart disease, left ventricular hypertrophy (LVH), atrial fibrillation (AF), and other related diseases were transcribed from various types of medical records including medical charts, lab reports, nursing diaries and reports of ECG, chest X-ray, and echocardiogram.

Blood samples of the patients were drawn after at least an 8-hour over-night fast. Fasting venous blood was drawn into two 5cc heparinized tubes. Plasma and buffy coat were prepared immediately after drawing and stored at -70°C. Heparinized plasma was used to measure total cholesterol (TC) and high-density lipoprotein cholesterol (HDL-C) (Lieberman-Burchard method), triglyceride (Bucolo method), and glucose (Keston method), with a Hitachi autoanalyzer (Hitachi 7250, Hitachi, Japan). Low-density lipoprotein cholesterol (LDL-C) value was calculated from levels of TC, triglyceride and HDL-C[33]. The coefficient of variation of 65 duplicated samples was 2.2% for TC, 3.1% for triglyceride, 2.8% for HDL-C and 2.5% for glucose. The stroke patients were followed from admission to 3 months later. In this study, we analyzed the data of the blood after 3 months of onset in stroke patients to ensure that blood levels were stabilized.

Hypertension was defined by systolic/diastolic blood pressure ≧140/90 mm Hg or by receiving antihypertensive therapy. The measurement of blood pressure from left arm was obtained and used. Diabetes was defined by fasting plasma glucose ≧126 mg/dL or by taking hypoglycemic medication. Patients with hypercholesterolemia, hypertriglyceridemia, high LDL-C level and low HDL-C level were defined by total cholesterol level ≧240 mg/dL, triglyceride level ≧200 mg/dL, TC/HDL-C ratio ≧5, LDL-C level ≧130 mg/dL, HDL-C level < 40 mg/dL (for men)/< 50 mg/dL (for women), respectively. Obesity was defined as body mass index ≧27. Ever-smoker was those subjects with current or past smoking habits.

An extracranial carotid duplex ultrasound machine (SONO 1000; Hewlett-Packard Company; USA), with a transducer frequency of 7.5 Hz and color frequency of 5.4 Hz, was used. A standardized protocol was established. The near and far walls of the left and right proximal common carotid artery (CCA), distal CCA, proximal external carotid artery, proximal internal carotid artery, and carotid bifurcation were examined by B-mode duplex scanning. The degree of plaque was graded as follows: 0 = no plaque; 1 = one small plaque < 30% of the vessel diameter; 2 = one medium plaque between 30% and 50% of the vessel diameter or multiple small plaques; 3 = one large plaque > 50% of the vessel diameter or multiple plaques with at least one medium plaque. The grades in each segment of all carotid arteries were added to create a summary plaque score corresponding to the extent of carotid atherosclerosis.

### Analysis of Length Variability of (GT)n Repeats in HO-1 Gene Promoter

Genomic DNAs were extracted from storaged leukocytes by conventional procedures. The 5'-flanking region containing (GT)_n _repeats of the HO-1 gene was amplified by PCR with a FAM-labeled sense primer, 5'-AGAGCCTGCAGCTTCTCAGA-3', and an antisense primer, 5'-ACAAAGTCTGGCCATAGGAC-3', according to the published procedure [34]. The PCR products were mixed together with GenoType™ TAMRA DNA ladder (size range 50-500 bp) (GibcoBRL) and analyzed with automated DNA sequencer (ABI Prism™ 377). Each size of the (GT)_n _repeat was calculated using the GeneScan Analysis software (PE Applied Biosystems).

### Statistical analysis

The distribution of the numbers of (GT) _n _repeats of two DNA strands were studied, and the frequency of repeats in patients was plotted. Assuming a co-dominant (additive) trait model, HO-1 genotypes were defined by the averaged length of (GT)_n _repeats. Averaged length of (GT) _n _repeats of the HO-1 gene promoter was calculated for each patient.

Age was expressed as mean ± SD and compared by Student's *t *tests. Chi-square test was used to examine whether CAD risk factors (hypertension, diabetes, all lipids abnormalities, and smoking habits) and some other characteristics differed between IS patients and NS controls. Chi-square test was also used to compare frequency of genotype S and L between IS patients and NS controls in all subjects or in subgroups stratified by above risk factors. However, only the figure on HDL-C was shown. The associations between stroke status and HO-1 genotypes were examined by stratifying on stroke and cardiovascular (CVD) risk factors. The risk of odds ratio (OR) was showed after adjusted the following factors: age, sex, hypertension, diabetes, smoking habits, lipid abnormalities, obesity, or plaque score if necessary. Two sides p value was calculated, and significant level was accepted at *P *< 0.05. Statistical calculations were performed using SAS software package (version 9.1).

## Results

**Figure 1:**
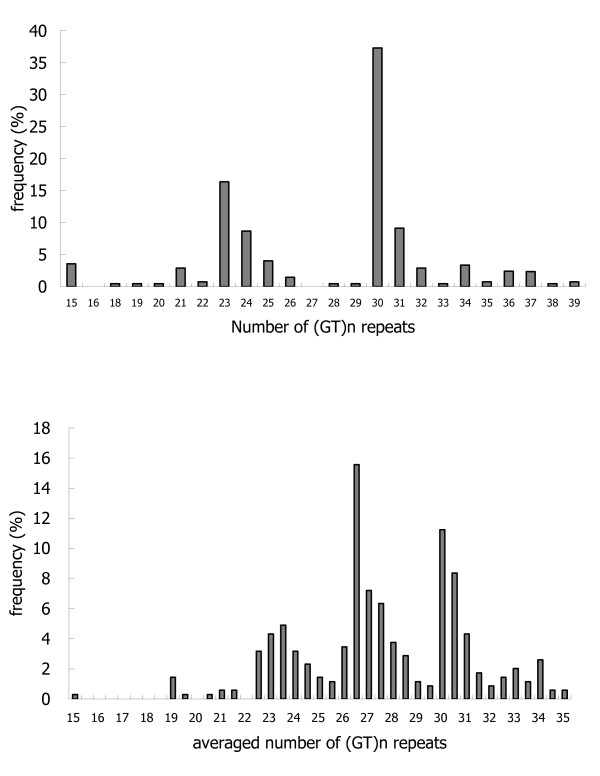
**Allele frequency distribution of (GT)n repeats and the averaged (GT)n repeats in the HO-1 gene in 347 patients**.

The length of the (GT)_n _repeats in the human HO-1 gene promoter region ranged from 15 to 39, as shown in figure 1. The distribution had two peaks at (GT)_23 _and (GT)_30_, respectively. The averaged length of the (GT)_n _repeats had similar distribution, but they ranged from15 to 35 with peaks at 27 and 30. Therefore, we defined genotype short (S) for those with averaged length ≦26 GT repeats, and genotype long (L) for those with length of >26 GT repeats, which included around 70 percent of patients.

Stroke patients were significantly older (mean aged 65.5 ± 12.2 versus 62.8 ± 11.5 in controls, p = 0.0465). They also had significantly greater proportion of diabetes, hypertension, LDL-C, lowered HDL-C level, obesity, and higher plaque score than the controls (Table 1). Additionally, stroke patients tended to have more males, more ever-smokers, more lipids abnormalities and more genotype L than controls. However, no significant differences were found between IS and NS patients.

**Table 1:** Distribution of CVD risk factor status in stroke patients and their controls

		Stroke patients (n = 183)	Non-stroke patients (n = 164)	
		
Variables		N (%)	N (%)	P
Gender	Male	114 (62.3)	87 (53.1)	0.0815
Diabetes	Yes^§^	64 (35.0)	19 (11.6)	<0.0001***
Hypertension	Yes^&^	151 (82.5)	90 (54.9)	<0.0001***
TC	≧240 mg/dL^+^	26 (14.2)	32 (19.5)	0.1861
Triglyceride	≧200 mg/dL^+^	44 (24.0)	38 (23.2)	0.8480
LDL-C	≧130 mg/dL^+^	52 (28.4)	66 (40.2)	0.0202*
HDL-C	<40/50 mg/dL in male/female	131 (71.6)	68 (41.5)	0.0001***
TC/HDL-C ratio	≧5^+^	66 (36.1)	58 (35.4)	0.8920
Smoker	Ever (current/past)	73 (39.9)	47 (28.7)	0.0804
BMI	≧27	23 (12.6)	40 (24.4)	0.0043**
Plaque score	≧3	83 (45.3)	43 (26.2)	0.0002***
Genotype	L: >26 GT repeats	138 (75.4)	114 (69.5)	0.4262

^§ ^fasting blood glucose ≧126 mg/dL or on DM medication. ^&^SBP ≧140 mmHg or DBP ≧90 mmHg or on anti-hypertension medication. ^+ ^included those patients with related medication. TC: total cholesterol; HDL-C: high-density lipoprotein cholesterol; LDL-C: low-density lipoprotein cholesterol; BMI: body mass index. P: the p value from chi-square test between stroke and non-stroke patients. Genotype; the length of GT repeats in HO-1 gene promoter. *: p < 0.05; **: p < 0.01; ***: p < 0.001

Table 2 shows the distribution of genotypes of HO-1 gene promoter by several cardiovascular risk factors, respectively, in stroke patients and in non-stroke controls. A higher proportion of genotype L was observed in stroke patients than in non-stroke patients in those with lowered HDL-C (p = 0.0056), normal TC level (p = 0.0490), and normal LDL-C level (p = 0.0270), so was in those with high TC/HDL-C ratio with borderline significant (p = 0.0747). Stroke patients tended to have higher frequency of genotype L than non-stroke patients in patients with diabetes, hypertension, hyperlipidemia, ever-smoking habit and high carotid plaque score, respectively; but these were not statistically significant (Table 2). In addition, stratified analysis further showed that diabetics tended to have higher proportion of genotype L than non-diabetics. But the significance was only shown for stroke patients (p = 0.0389).

**Table 2:** Distribution of genotype of HO-1 gene promoter by risk factor status in stroke patients and their controls

		Stroke patients (n = 183)		Non-Stroke patients (n = 164)		
		
Variables	Groups	S	L	S	L	P
All subjects		45 (24.6)	138 (75.4)	50 (30.5)	114 (69.5)	0.4262
Hypertension^&^	Yes	37 (24.5)	114 (35.5)	26 (28.9)	64 (71.1)	0.4535
	No	8 (25.0)	24 (75.0)	24 (32.4)	50 (67.6)	0.4441
Diabetes *	Yes	10 (15.6)	54 (84.4)	4 (21.1)	15 (79.0)	0.8531
	None	35 (29.4)	84 (70.6)	46 (31.7)	99 (68.3)	0.8824
TC	≧240 mg/dL^+^	9 (34.6)	17 (65.4)	6 (18.8)	26 (81.3)	0.2310
	Otherwise	36 (22.9)	121 (77.1)	44 (33.3)	88 (66.7)	0.0490 *
Triglyceride	≧200 mg/dL^+^	11 (25.0)	33 (75.0)	13 (34.2)	25 (65.8)	0.4664
	Otherwise	34 (24.5)	105 (75.5)	37 (29.4)	89 (70.6)	0.3680
LDL-C	≧130 mg/dL^+^	15 (28.9)	37 (71.2)	14 (21.2)	52 (78.8)	0.3389
	Otherwise	30 (22.9)	101 (77.1)	36 (36.7)	62 (63.3)	0.0270 *
HDL-C	<40/50 mg/dL in male/female	31 (23.7)	100 (76.3)	29 (42.7)	39 (57.4)	0.0056 **
	Otherwise	14 (26.9)	38 (73.1)	21 (21.9)	75 (78.1)	0.4902
TC/HDL-C ratio	≧5^+^	14 (21.2)	52 (78.8)	21 (36.2)	37 (63.8)	0.0747
	Otherwise	31 (26.5)	86 (73.5)	29 (27.4)	77 (72.6)	0.8758
smoker	Current + past	17 (23.3)	56 (76.7)	12 (25.5)	35 (74.5)	0.8288
	Otherwise	28 (25.5)	82 (74.6)	38 (32.5)	79 (67.5)	0.3060
BMI	≧27	6 (26.1)	17 (73.9)	11 (27.5)	29 (72.5)	0.9032
	otherwise	38 (24.4)	121 (75.6)	39 (31.4)	85 (68.6)	0.1851
Plaque score	≧3	19 (22.8)	64 (77.1)	10 (23.3)	33 (76.7)	0.9633
	<3	26 (26.0)	74 (74.0)	40 (33.1)	81 (66.9)	0.3018

N(%). * fasting blood glucose 126 mg/dL or on DM medication. ^&^SBP 140 mmHg or DBP 90 mmHg or on anti-hypertension medication. ^+ ^included those patients with related medication. TC: total cholesterol. HDL-C: high-density lipoprotein cholesterol; BMI: body mass index. P: the p value from chi-square test comparing genotype S and genotype L between stroke and non-stroke patients in each CVD characteristic subgroup. S and L genotypes: averaged (GT)n repeats 26 and >26, respectively. *: p < 0.05; **: p < 0.01; ***: p < 0.001

**Figure 2:**
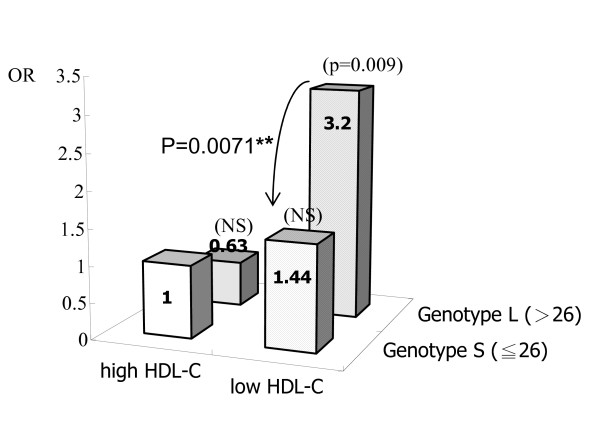
**Age- and sex- adjusted odds ratio on stroke risk by HO-1 genotypes by HDL-C level**. Low HDL-C status is HDL-C level less than 40 mg/dL in men or 50 mg/dL in women. P-values in parenthesis were obtained for each subgroup comparing with the reference group (low risk and genotype S), and those p values of ≧0.05 were not showed. **Bold **p value near curved arrow was obtained for comparing risk of genotype L with S in the high risk factor profile group. NS: p ≧0.05. *: p < 0.05. **: p < 0.01. ***: p < 0.001

According the results of table 2, we next examined whether the genotype of the human HO-1 gene promoter was associated with ischemic stroke under different lipids conditions: normal TC level and LDL-C level as well as abnormal HDL-C level (Table 3). In analysis of these subgroup, only the significant adjusted ORs were showed in those with lowered HDL-C levels (Model I and II OR & p value, 2.07, 0.0303 and 2.02, 0.0405, respectively). The multivariate ORs were not significant in the analyses of TC and LDL-C. For describing the interaction of HDL-C and genotypes, figure 2 shows the age- and sex- adjusted OR on stroke risk for HO-1 gene genotypes by the HDL-C status. Patients of genotype L tended to have larger ORs than those of genotype S in people carrying lowered HDL-C status (genotype L vs S in OR: 3.20 vs 1.44 in low HDL-C group), as well as other CVD risk factors (detailed data not shown). Similar trend was found but with no statistical significance. In addition, we also examined the increased effects for genotype L in comparing with S in each high risk factor profile group. In those with low HDL-C, subjects with genotype L had significantly greater stroke risk than those with genotype S (p = 0.007).

**Table 3:** OR on stroke risk of genotype L compared to genotype S in 3 subgroup analyses

		Model I			Model II		
		**OR**	**(95%CI)**	**P**	**OR**	**(95%CI)**	**P**

HDL-C	<40/50 mg/dL in male/female	2.07	(1.07-4.01)	0.0303	2.02	(1.01-4.02)	0.0465
TC	< 240 mg/dL	1.62	(0.93-2.83)	0.0918	1.63	(0.88-2.39)	0.1182
LDL-C	< 130 mg/dL	1.91	(1.01-3.61)	0.0480	1.63	(0.85-3.13)	0.1435

Odds Ratio (95% confidence interval). TC: total cholesterol. HDL-C: high-density lipoprotein cholesterol. P: the p value from logistic regression comparing the genotype L versus genotype S (reference) on stroke risk in each CVD characteristic subgroup. S and L genotypes: averaged (GT)n repeats ≦26 and >26, respectively. Model I: adjusted for age, sex and plaque score; Model II: adjusted for age, sex, hypertension, diabetes, ever-smoking, body mass index ≧27, and plaque score, as well as hyperlipidemia or hypo-HDL-Cholesterolemia depends.

## Discussion

Our study revealed that shorter HO-1 promoter genotype has the protective effect on ischemic stroke, especially in the patients with lower HDL-C levels. The effects and interactions were demonstrated in univariate and multivariate models for all subjects, and the protective gene effect appeared in the stratified high risk group, not in the low risk group. The significant increases of genotype L than S indicated the protective genetic effects of shorter HO-1 gene repeats seem work up well in high risk group like low HDL-C level, and the risk was decreased to the level near the low risk group like appropriate HDL-C level. All of patients in our study had no prior CAD or stroke, so our observation was not confounded by selection bias, such as survivor for severe disease. It is less considered in previous studies.

Interestingly, we also described more risk effects of genotype L on stroke in those with normal TC or LDL-C levels than those of genotype S, although the effect was no longer present in multivariate analysis. Before our study, one study reported shorter repeats in HO-1 gene exerted a protective effect on the development of ischemic cerebrovascular events definitely in patients without hypercholesterolemia [3]. The finding was similar with that in our study, but they did not include HDL-C measure. In summary of our findings, the protective effect of HO-1 genotype on ischemic stroke depended on the presence of lipid conditions, that is, the levels of HDL-C, it may explain the controversial findings in the literatures.

Similar as the previous CAD studies, we did not find the significant difference of averaged (GT)_n _repeats in HO-1 gene promoter between IS and NS patients. Instead, the lengths of (GT)_n _repeat seem associated with the ischemic stroke status only in those individuals with lowered HDL-C level from our observation and some previous studies. Chen and his coauthors have shown a protection of HO-1 genotype in diabetic CAD patients with at least 1 coronary narrowing (>75%) and in restenosis patients after coronary stenting [24,35]. Another study has shown that the longer (GT)_n _repeat in HO-1 gene promoter was related to CAD risk in diabetic and hypercholesterolemic patients [25]. Interestingly, in the other study, HO-1 gene exerted a protective effect on ischemic cerebrovascular events in patients with normal cholesterol level [3]. No effect of HO-1 genotype could be observed in total population, they only have been observed in high risk group or low risk group. It implies that long (GT)_n _repeats alone may not be sufficient to cause diseases. But it may contribute to the development of the disease when certain conditions of enhanced oxidative stress coexist, including the abnormal composition of cholesterol levels in patients with normal total cholesterol levels.

Many studies reported that HO-1 gene involved in the mechanism against the development of atherosclerosis. Animal studies reported that products of HO pathway such as bilirubin act as a significant protective factor for atherosclerosis[27]. The modulation of HO-1 gene expression in LDL-C receptor-deficient mice influence the progression of atherosclerosis [17], and mice treated with the HO-1 inducer exhibited reduced atherosclerotic lesion formation. Vascular proliferation was inhibited by transferring HO-1 gene[18]. These observations support that HO-1 functions as an intrinsic protective factor against atherosclerotic lesion formation and may be an anti-atherogenic role in vascular wall [15].

Chen et al conducted a transient transfection experiment in rat aortic smooth muscle cells to show that longer (GT)_n _repeats in HO-1 promoter decreased luciferase promoter activity, indicating decrease in gene transcription in vascular cells[24]. They also found that genotype L/L carriers are associated with higher extent of sever lipid peroxidation, supporting the genetic influence of HO-1 on oxidative stress. Lipid abnormalities like hypercholesterolemia correlate with enhanced oxidative stress. HDL-C acts as an anti-oxidant through its capability of inhibiting LDL-C oxidation, preventing the formation of lipid hydroperoxides [36,37]. Excessive oxidative stress was considered as a potential cause of the vascular disease and other complications in hyperglycemic patients [24,38]. S genotype in the HO-1 gene promoter may increase the induction of HO-1 by reactive oxygen species in patients with low HDL-C concentrations. The insufficient effect of anti-oxidative stress due to lower HDL-C levels may be reversed by S genotype in HO-1 gene promoter, thereby reducing the risk of cerebral ischemia.

In this study, the allelic frequency distribution of the lengths of (GT)_n _repeats in the HO-1 promoter in recruited subjects (range from 15 to 39) was similar with that in the previous reports[23-25,34,35]. The previous studies defined L and S alleles first and constructed genotypes SS, SL, and LL to examine the disease risk. We demonstrated the results assuming co-dominant (additive) model and using averaged length of two alleles to define genotype L and S, since the latter is more powerful and fits well the characteristics of complex model. We also obtained consistent results using the traditional (former) classification method: the age, sex & plaque score-adjusted OR of 2.26 (p = 0.0263) and multivariate OR of 1.82 (p = 0.0691) in those with lower HDL-C level while those carriers with homozygous S allele (≦26 GT repeats) as genotype SS compared with otherwise. The borderline significant findings on HO-1 genotype and cerebral ischemia were found in those with low HDL-C level or in those with high TC/HDL-C ratio. Therefore, the role of HDL-C associated with developing ischemic stroke exists identically but underestimated. Such finding focused the additive effect implied the equal importance in double helix, then decreasing number of GT repeats act additively with the increasing protective effects, as some previous reports described[39,40].

The limitations of this study should be mentioned. Our study is a case control study. The controls were recruited from outpatients of the same hospital who seemed to have higher levels of several CVD risk factors than general population. On the other hand, although we only included the first case without the history of CAD or stroke to reduce the selection bias, however, we still not avoid the loss in the stroke patients died before admission. Fortunately, the number is few. Taken together, the effect of HO-1 genotype on ischemic stroke may have been underestimated.

## Conclusions

We have demonstrated that the long lengths of (GT)_n _repeats in HO-1 gene promoter are associated with the high risk status on cerebral infarction in subjects with low HDL-C status. The protection form shorter HO-1 gene promoter on stroke may be more critical in patients with lower HDL-C levels than in those with higher HDL-C levels. The findings suggest that genetic characteristics of the HO-1 gene may interact with the oxidative stress conditions to contribute to the development of ischemic stroke. These findings should be confirmed further in population-based studies.

## Competing interests

The authors declare that they have no competing interests.

## Authors' contributions

CHB participated in the design of the study, carried out the data collection from interview and lab, performed the statistical analysis, drafted and revised the manuscript. JRC and HCC carried out the screen and enrolment of all patients (cases and controls), particularly by neurological evidences. CCC extracted all of the clinical information, and reconfirmed the diagnosis of chronic diseases such as dyslipidemia. LYC carried out all of the molecular genetic studies. WHP conceived of the study, and participated in its design and coordination. All authors read and approved the final manuscript.
